# 78. Non-Invasive Prediction of Invasive Fungal Infection by Plasma-Based Microbial Cell-Free DNA Next-Generation Sequencing (mcfDNA NGS) in Pediatric Patients with Relapsed or Refractory Leukemia

**DOI:** 10.1093/ofid/ofab466.078

**Published:** 2021-12-04

**Authors:** Joshua Wolf, Joshua Wolf, Gabriela Maron, Kathryn Goggin, Kim J Allison, Pamela Merritt, Amanda griffen, Jeffrey Rubnitz, Radha Duttagupta, Abigail Brenner, Lily Blair, Asim A Ahmed, Cara Morin

**Affiliations:** 1 St. Jude’s Children’s Research Hospital, Memphis, TN; 2 St. Jude Children's Research Hospital, Memphis, Tennessee; 3 Emory University, Atlanta, Georgia; 4 Karius Inc, Redwood, California; 5 Indiana University, Indianapolis, Indiana; 6 Karius Inc., Redwood, California; 7 Karius, Inc, Redwood City, CA

## Abstract

**Background:**

Diagnosis of invasive fungal infections (IFIs), a life-threatening complication of cancer therapy or hematopoietic cell transplantation (HCT) can be challenging, and IFI has poor outcomes. Prediction or early non-invasive diagnosis of IFI in high-risk hosts *before* onset of symptoms could reduce morbidity and mortality.

Because non-invasive plasma mcfDNA NGS can detect invasive fungal infections, and may predict bloodstream infections in immunocompromised patients, we hypothesized that mcfDNA NGS might also predict invasive fungal infection before clinical presentation.

**Methods:**

In a prospective study, serial remnant plasma samples were collected from pediatric patients undergoing treatment for relapsed or refractory leukemia. IFI events were classified according to EORTC criteria by 2 independent experts, and episodes empirically treated for suspected IFI, but not meeting ‘possible’ criteria were classified as ‘suspected’. All samples collected within 30 days before clinical diagnosis of non-fungemic IFI were tested for fungal DNA by mcfDNA NGS using a research-use only assay by Karius, Inc. optimized for fungi; because of overlapping clinical syndromes, non-fungal DNA was not considered in this study.

**Results:**

There were 15 episodes of suspected IFI in 14 participants with ≥1 sample available from either diagnostic (within 1 day of diagnosis) or predictive (2 to 30 days prior to diagnosis) periods (5 “suspected”, and 4 probable and 6 proven by EORTC definitions).

Of 10 probable or proven IFIs, 6 (60%) had a relevant fungal pathogen identified mcfDNA NGS at diagnosis. In each of these cases the fungal DNA was also detectable prior to clinical onset of IFI (Range 2 to 41 days; Figure 1). In an additional case, manual review of sequence data identified the fungal DNA at diagnosis and during the prior month. Of 5 “suspected” IFI episodes, all were determined by expert review as not representing fungal infection; fungal DNA was identified by mcfDNA NGS in 2/54 (3.7%) of samples from these episodes.

Table 1. Characteristics of Invasive Fungal Infections

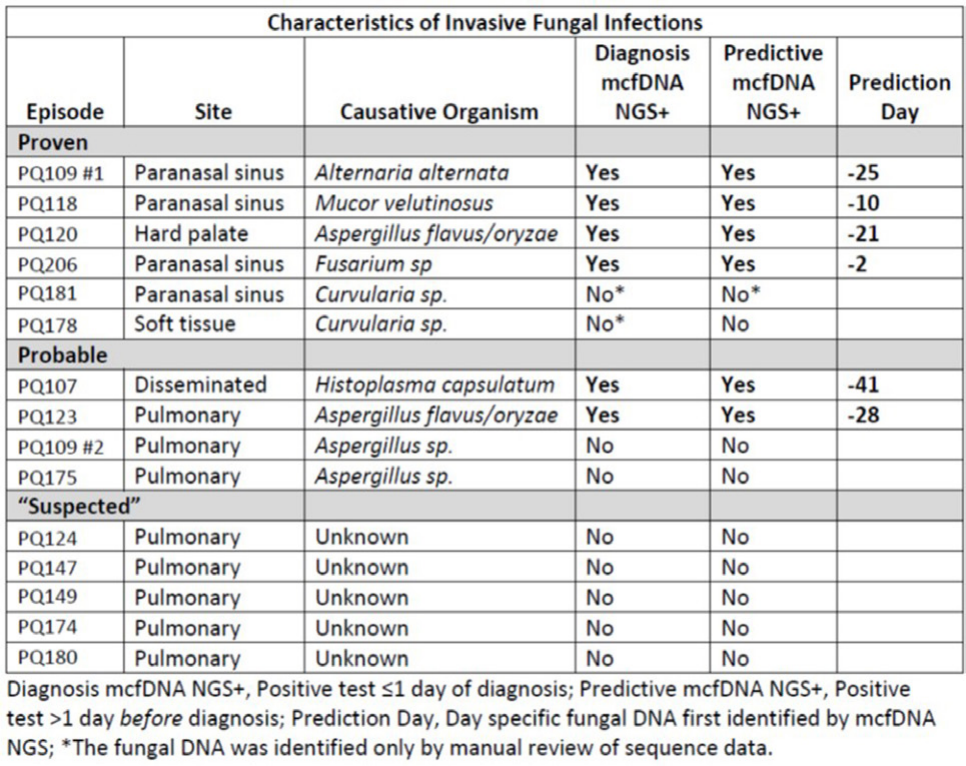

**Conclusion:**

mcfDNA NGS can identify fungal pathogen DNA before clinical onset of IFI, so might predict IFI in immunocompromised hosts, and may help differentiate fungal infection from other etiologies of lung nodules or infiltrates.

**Disclosures:**

**Joshua Wolf, MBBS, PhD, FRACP**, **Karius Inc.** (Research Grant or Support) **Joshua Wolf, MBBS, PhD, FRACP**, Nothing to disclose **Radha Duttagupta, PhD**, **Karius inc** (Employee) **Lily Blair, PhD**, **Karius Inc.** (Employee) **Asim A. Ahmed, MD**, **Karius, Inc.** (Employee)

